# Health care waste management: a multi speed development in the sub-Sahara African region

**DOI:** 10.11604/pamj.2014.17.305.3965

**Published:** 2014-04-22

**Authors:** Jean Gerard Tatou Doumtsop

**Affiliations:** 1 Ministry of Public health, Cameroon

**Keywords:** Health care, waste management, Africa

## Editorial

Having visited many health care facilities in some countries of sub-Sahara African region, I have come to notice that alongside often insurmountable difficulties by health personnel per se such as water supply or electricity supply lay an important rather more surmountable problem of health care waste (HCW) management. In 2002, WHO assessment in 22 developing countries showed that 18-64% of Health care facilities do not use proper waste disposal methods [1]. No doubt that the number of health care facilities has increased significantly over the last decade due to demographic development but mainly to global aid from international commitment to eradicate and control many diseases ( Global funds, Clinton Health Access Initiative, GAVI alliance, C2D etc.) making the issue a serious rising problem. In my touring in different health facilities, Countless time I have forked here and there in order to slip away heaps of HCW within hospital enclosure from the top up reference hospital to the top down health post. When I have sometimes asked how does it comes to be like that, the most frequent answer has been that there is either no incinerator or that the one existing is broken down! I have asked myself about the common sense of health workers in our region. If we can say that a nurse in a health post is not well trained to intuitively dispose HCW in a safer manner, how does it come that even in some teaching hospital where there are highly educated health personnel there is still little care about safely disposal of HCW! Commonly, heaps of wastes are being burned here and there but rarely completely burned! If 15 to 25% of total HCW generated was potentially dangerous by the year 2000 [2], the rapid scaling up of activities such as Expanded Program of Immunization and other injection practices highly generator of potentially dangerous waste have likely increased that percentage very well.

I noticed in approximate same conditions that some reference hospitals either in the same country or between countries were performing better than others and I suspected some hypothetical reasons: The overall performance of the managing board; The overall concern of health workers about public health issues; The poor motivation to care about public goods as compare to private ones; The socio-economic standards of the catchment area of the health care facility; The number of Non Governmental Organisations working on health performing in the catchment area of the health care facility.

The concern about biomedical waste management was seriously considered by WHO in 2000 and grounds for good management laid down [3], but over the following decade the scale of the phenomenon in our sub-Sahara African region has not been really evaluated! The little concern of public health operational researchers in Africa may be due to the sensibility of the issue thus impairing them to easily obtain ethical clearance from decision makers, these latter, somehow more concerned with their reputation. Principles and methods from the simplest to the more sophisticated ones for biomedical waste management are well established and continue to be updated by interested organisations [4], but I still believe that for our very low income region, the challenge should be first what to do and how to do before what to use and how to use given our very low capacity to sustain a rapidly growing technology. As long as I remember that the problem was never mentioned about during our medical training period I also strongly believe that the best way to promote best practices in the long run is to include the matter in the training program in health schools. By the time they become health workers, their awareness about HCW management will certainly be better raised and they could intuitively apply corresponding best practices to their working setting. However, as developed by WHO [5], good HCW management definitely depends on a dedicated waste management team, good administration, careful planning, sound organisation underpinning legislation, adequate financing, and full participation by trained staff.
